# Targeting Hypercalciuria in SLC34A1-Related Disorders: Impact of Oral Phosphate Therapy and Novel Genetic Insights in Pediatric Case Series

**DOI:** 10.1007/s00223-025-01462-x

**Published:** 2026-01-16

**Authors:** Ihsan Turan, Muge Atar, Mehmet Eltan, Ahmet Anik, Eda Celebi Bitkin, Semine Ozdemir Dilek, Mevra Cay, Sevcan Tuğ Bozdogan, Hakan Döneray, Damla Kotan, Serap Turan, Bilgin Yüksel, Ali Kemal Topaloglu

**Affiliations:** 1 Division of Pediatric Endocrinology, Faculty of Medicine, Cukurova University, Adana, Turkey; 2 Division of Pediatric Endocrinology, Antalya Training and Research Hospital, Antalya, Turkey; 3 Division of Pediatric Endocrinology, School of Medicine, Marmara University, Istanbul, Turkey; 4 Division Pediatric Endocrinology, Adnan Menderes University School of Medicine, Aydin, Turkey; 5 Division of Pediatric Endocrinology, Bakırkoy Dr Sadi Konuk Training and Research Hospital, Istanbul, Turkey; 6 Department of Medical Genetics, Faculty of Medicine, Cukurova University, Adana, Turkey; 7 Division of Pediatric Endocrinology, Faculty of Medicine, Atarturk University, Erzurum, Turkey; 8 Division of Pediatric Endocrinology, Harvard Medical School, Massachusetts General Hospital, Boston, MA USA

**Keywords:** *SLC34A1*, Infantile hypercalcemia, Hypercalciuria, Hypophosphatemia

## Abstract

Pathogenic variants in the *SLC34A1* gene, which encodes the sodium-phosphate cotransporter NaPi-IIa, lead to a spectrum of renal tubular disorders, including infantile hypercalcemia type 2, nephrolithiasis/osteoporosis-hypophosphatemia type 2, and Fanconi renotubular syndrome type 2. Despite increasing recognition of *SLC34A1*-related disorders, data on genotype–phenotype correlations and treatment response remain limited due to the rarity of the condition. We retrospectively analyzed the clinical, biochemical, and molecular features of 11 patients from unrelated families carrying 12 pathogenic or likely pathogenic *SLC34A1* variants, three of which were novel. Next-generation sequencing and ACMG-AMP criteria were used for variant classification. Biochemical parameters including serum phosphate, calcium, parathyroid hormone, urinary calcium, and TmP/GFR were evaluated. Treatment response to oral phosphate supplementation was longitudinally assessed. All patients exhibited hypercalciuria and nephrocalcinosis at diagnosis. Oral phosphate supplementation (5–20 mg/kg/day) resulted in normalization of urinary calcium excretion in 10 of 11 cases, regardless of baseline serum phosphate status. Linear growth improved in all but one patient. The identified mutations clustered primarily within functional domains of the NaPi-IIa protein, particularly amino acid residues 109–205 and 375–487. Several splice-site and codon-specific variants—such as those affecting Gly153 and Gly194—were highlighted as potential pathogenic hotspots. Our findings expand the mutational and phenotypic spectrum of *SLC34A1*-related disease and reinforce the utility of oral phosphate supplementation in managing hypercalciuria and promoting growth. Functional domain mapping and variant clustering analyses enhance understanding of disease mechanisms and support the importance of early diagnosis and long-term surveillance.

## Introduction

Renal phosphate reabsorption is primarily mediated by the sodium-dependent phosphate transporters NaPi-IIa (*SLC34A1*) and NaPi-IIc (*SLC34A3*) [1]. *SLC34A1* encodes the renal sodium-phosphate cotransporter type IIa (NaPi-IIa), an electrogenic, sodium-dependent phosphate transporter located in the brush border membrane of renal proximal tubules [2]. Pathogenic variants in the *SLC34A1* gene may cause a range of clinical phenotypes, including infantile hypercalcemia type 2 (IH2) (Omim: 616,963), nephrolithiasis/osteoporosis-hypophosphatemia type 2 (NPHLOP2) (Omim: 612,286), and Fanconi renotubular syndrome type 2 (Omim: 613388) [3, 4]. Patients harboring *SLC34A1* mutations typically present with hypophosphatemia and hypercalciuria. Renal phosphate wasting leads to decreased serum phosphate levels, which in turn suppresses fibroblast growth factor 23 (FGF23). The reduction in FGF23 stimulates the synthesis of 1,25-dihydroxyvitamin D₃ [1,25(OH)₂D₃] by upregulating renal 1α-hydroxylase (CYP27B1) and concurrently reducing its catabolism via 24-hydroxylase (CYP24A1). Elevated 1,25(OH)₂D₃ levels subsequently enhance intestinal absorption of both phosphate and calcium. The excess calcium is excreted by the kidneys, leading to hypercalciuria and an increased risk of nephrolithiasis and nephrocalcinosis [5].

Renal calcification, caused by long-term hypercalciuria, is a significant manifestation in these cases [4, 6, 7]. Furthermore, *NPT2a* has been associated with nephrolithiasis and altered renal function in genome-wide association studies [7–10]. Owing to the rarity of the condition, data on its clinical management and genotype–phenotype correlations remain limited [11, 12]. In this study, we present the clinical and genetic features of 11 patients who carry SLC34A1 pathogenic variants and analyze their response to treatment.

## Methods

In this multicenter retrospective case series, we analyzed the clinical phenotype and genetic profile of 11 pediatric probands carrying pathogenic variants in *SLC34A1*; all molecular evaluations were performed using next-generation sequencing. The reference transcript used for *SLC34A1* was NM_003052.5. Identified variants were assessed using the Human Gene Mutation Database (HGMD), PubMed, ClinVar, and Google Scholar. Previously reported variants were appropriately cited. The minor allele frequency (MAF) for each variant was determined using the Genome Aggregation Database (gnomAD) Non-Finnish European [13, 14] and the Turkish variome [15]. All variants were subsequently classified according to the 2015 guidelines of the American College of Medical Genetics and Genomics and the Association for Molecular Pathology (ACMG–AMP) using the Franklin platform [16]. Pathogenicity criteria were very strong (PVS1), strong (PS1–PS4), moderate (PM1–PM6), or supporting (PP1–PP5), and benign criteria stand-alone (BA1), strong (BS1–BS4), or supporting (BP1–BP7) were noted for each candidate variant.

Height standard deviation scores (SDS) at presentation were calculated for all patients using national growth curves for Turkish children [17]. Urinary calcium and phosphorus excretion were calculated from spot urine samples. Renal tubular reabsorption of phosphate (TRP and TmP/GFR) was calculated (TRP = 1–[(Up/Pp) × (Pcr/Ucr)]TmP/GFR = Pp–(Up/Ucr) × Pcr, where Up denotes urinary phosphate, Pp plasma phosphate, Pcr plasma creatinine, and Ucr urinary creatinine [18]. Reference ranges of calcium, phosphorus, alkaline phosphatase, and TmP/GFR were used in cited articles [18, 19]. Nephrocalcinosis was determined by renal ultrasonography. The Wilcoxon signed-rank test was used to compare spot urinary calcium/creatinine values before and after phosphorus treatment. The study protocol was approved by the Ethics Committee of Çukurova University Faculty of Medicine.

## Results

**Fig. 1:**
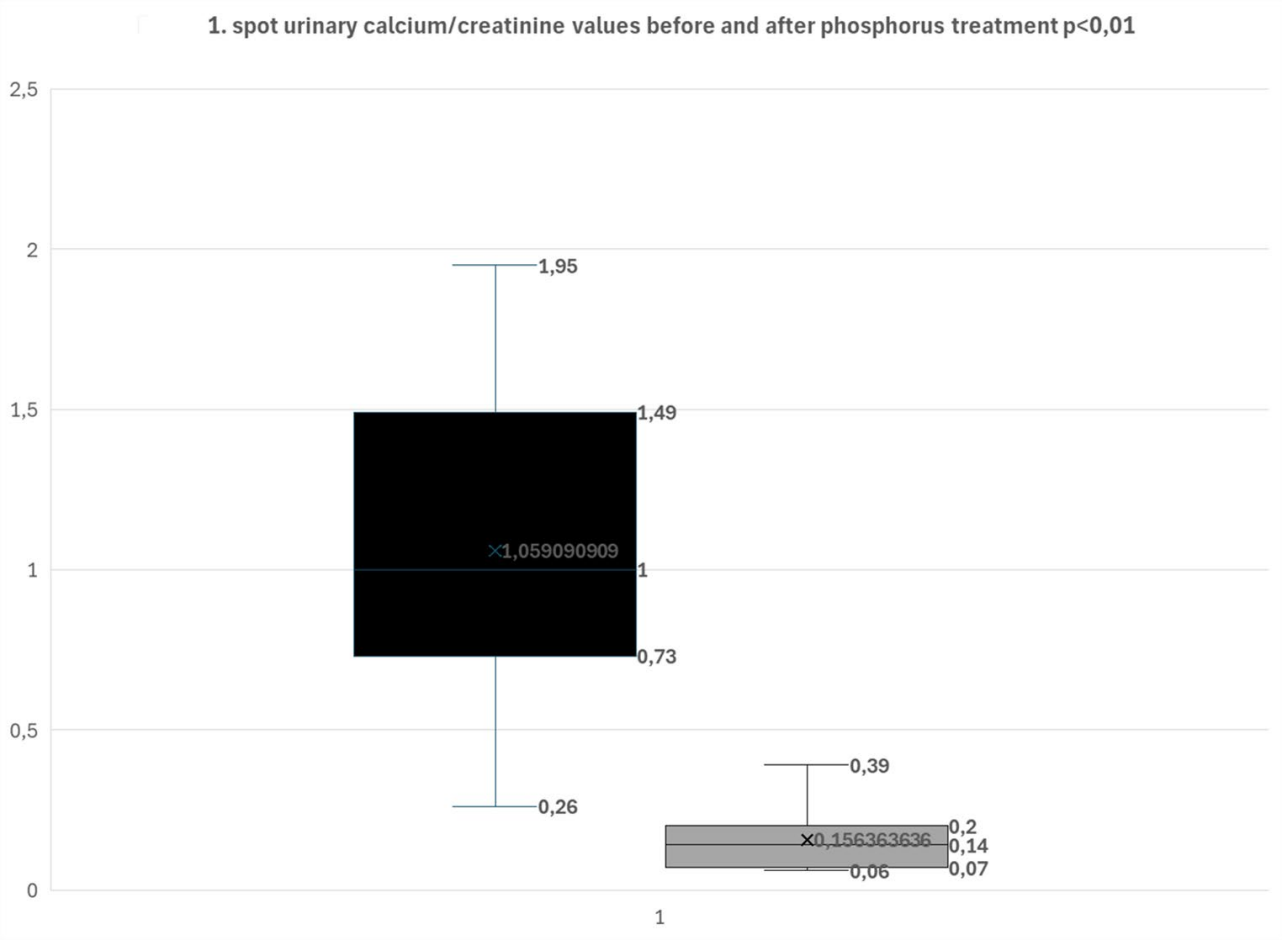
Urinary calcium levels before and after phosphate supplmentation

The case series comprised 11 patients, 8 of whom were male (72%); median age at diagnosis was 3 months (1 month–14 years). At diagnosis, the median age was 4 months, and 16 months in biallelic variant carriers and heterozygous cases, respectively. The most frequent presenting feature was failure to thrive (Patients 3, 4, 5, and 11). In three cases (Patients 6, 8, and 9), nephrocalcinosis identified on renal ultrasonography—performed for unrelated indications (urinary tract infection, prenatal screening, or incidentally)—prompted diagnostic evaluation. Two patients (Patients 1 and 2) presented with irritability/restlessness, and one (Patient 10) was identified due to hypercalcemia detected during hospitalization for pneumonia. Patient 7 presented with genu valgum. Bone radiographs showed no evidence of rickets in any patient; no additional skeletal abnormalities were identified, apart from genu valgum in Patient 7. All patients presented with hypercalciuria and/or nephrocalcinosis at diagnosis. Notably, hypercalciuria improved in all patients following oral phosphate supplementation, even when serum phosphate levels were within normal limits, indicating efficacy of phosphate therapy irrespective of baseline serum phosphate concentrations. The decrease in urinary calcium/creatinine ratio after phosphorus treatment was statistically significant (p = 0.001631). Additionally, nephrocalcinosis resolved in two patients (Patient # 9 and #11) during follow-up with treatment. No other shared findings were identified among the case presentations. Table 1 summarizes the clinical and laboratory characteristics of the cohort and delineates the association between phosphate supplementation and the occurrence of hypercalciuria Fig. 1.

**Table 1:** Summarizes the clinical and laboratory characteristics of the cohort

Patient	Age	Gender/presentation	Height/weight SDS	Serum calcium mg/dL	Serum phosphorus mg/dL	ALP U/L	TmP/GFRmmol/L	TRP (%)	PTH (pg/mL) (14–65)	25-OH-D3 ng/mL (20–80)	1,25-(OH)2D3 pg/mL (F:18–78 M:18–64)	sU-Ca/Cre	Renal USG	Association between phosphor treatment hypercalcemia
1^PV^	5m	F/restlessness	− 1,48/ − 0,15	13.3 (9–10,6)	3,0 (5,2–8,4)	272 (122–469)	0,76 (1,48–3,30)	77	1,2	23,15	26	0,83 (< 0,8)	NC	HC resolved following phosphorus supplementation initiated at the initial visit. The treatment was subsequently self-discontinued, and no recurrence of HC to date
1^LV^	3y7m		− 1.75/− 0.66	9.6 (8,8–10,8)	4,2 (4,5–6,5)	198 (142–335)	NA	NA	NA	NA	NA	0,18 (< 0,2)	NC	
2^PV^	1m	M/jaundice and restlessness	− 3.57/− 3.55	13,4 (9–10,6)	4,5 (5,2–8,4)	408 (122–469)	1,60 (1,43–3,43)	93	0,7	21	53	1,23 (< 0,8)	NC	HC initially resolved with phosphorus supplementation following the first clinical visit. However, after self-discontinuation of treatment, HC recurred sU-Ca/Cre: 2,2 and subsequently resolved with re-initiation of phosphorus therapy at a dose of 5 mg/kg/day
2^LV^	6y4m		− 0.5/− 0.83	8.8 (8,8–10,8)	3.6 (3,6–5,8)	382 (129–417)	1.16 (1,15–2,44)	99	24.8	29.1	NA	0,2 (< 0,2)	NC	
3^PV^	2y3m	M/failure to thrive	NA	10.0 (8,8–10,8)	2.4 (4,5–6,5)	626 (142–335)	0.63 (1,15–2,44)	82	21.0	6.9	44	0,73 (< 0,2)	NC	HC regressed following the initiation of phosphate supplementation after the initial clinical evaluation sU-Ca/Cre: 0,28 (< 0,2),
3^LV^	5y5m		− 3.09/− 2.64	9.48 (8,8–10,8)	2.6 (3,6–5,8)	925 (142–335)	NA	NA	37.0	11	NA	0,10 (< 0,2)	NC	
4^PV^	5m	F/failure to thrive, polyuria	− 2.45/− 2.63	15.0 (9–10,6)	2.8 (5,2–8,4)	168 (122–469)	0.58 (1,48–3,30)	96	0.1	57.7	75	1,52 (< 0,7)	NC RPVC	HC resolved following the initiation of phosphate supplementation after the initial clinical evaluation. However, recurrence occurred after the family discontinued treatment (sU-Ca/Cre: 0,29 (< 0,2). Reintroduction of phosphate therapy at a dose of 20 mg/kg/day subsequently led to resolution of HC
4^LV^	5y3m		− 1.98/− 2.39	10.8 (8,8–10,8)	3.5 (3.6–5.8)	164 (142–335)	0,78 (1.15–2.44)	68	44.0	NA	NA	0,06 (< 0,29)	Normal	
5^PV^	0y4m	M/failure to suck and thrive, polyuria	0/− 1.97	13.8 (9–10,6)	2.0 (5,2–8,4)	251 (122–469)	NA	97	8.4	NA	NA	0,78 (< 0,8)	NC	HC resolved following the initiation of phosphate supplementation after the initial evaluation (sU-Ca/Cre: 0,2 (< 0,2). Hc recuured even after discontinuation of treatment (sU-Ca/Cre: 0,26 (< 0,2)
5^LV^	5y11m		0.19/− 0.43	10.7 (8,8–10,8)	4.7 (4,5–6,5)	193 (142–335)	NA	NA	NA	NA	NA	0,26 (< 0,2)	NC	
6^PV^	0y3m	M/renal pathology (intrauterine usg)—> increased renal parenchymal echogenicity (newborn usg)	− 0.35/− 1.19	12.9 (9–10,6)	3.8 (5,2–8,4)	237 (122–469)	0.67 (1,48–3,30)	93	0.1	33	38	1,37 (< 0,8)	NC	HC resolved with phosphate supplementation at a dose of 10 mg/kg/day. The patient’s mother has a documented history of nephrolithiasis
6^LV^	1y1m		− 2.5/− 2.1	10.8 (9–10,6)	4.1 (4,5–6,5)	255 (142–335)	NA	NA	3.2	42	NA	0,39 (< 0,6)	NC	
7^PV^	14y	M/Genu valgum	− 3.08/− 2.44	9.6 (8,8–10,8)	1.9 (2,3–4,5)	371* (116–468)	0.33 (1,15–2,44)	47	NA	15	NA	0,49 (< 0,2)	NL: 2mm	There is evidence of treatment noncompliance; however, HC regressed with phosphate supplementation (sU-Ca/Cre: 0,16 (< 0,2). Notably, NC was not detected at any time during follow-up
7^LV^	21y		− 2.63/− 1.42	10.0 (8,8–10,8)	2.5 (2,3–4,5)	247 (55–149)	0.60 (0,96–1,44)	73	27.7	11	71	0,33 (< 0,2)	NL: 6 and 4,5 mm	
8^PV^	0y10m	M/urinary tract infection	0.62/− 0.43	11.5 (9–10,6)	4.7 (5,0–7,8)	230 (122–469)	1.38 (1,15–2,60)	90	7.1	10.8	40.4	1.00 (< 0,6)	NC	HC resolved following the initiation of phosphate supplementation after the initial clinical assessment
8^LV^	5y2m		0.31/− 0.66	9.9 (8,8–10,8)	5.8 (3,6–5,8)	207 (142–335)	1.49 (1,15–2,44)	79	21.6	33.9	NA	0,07 (< 0,2)	NC	
9^PV^	0y5m	M/Nephrocalcinosis on renal USG	− 0.64/− 1.48	10.6 (9–10,6)	4.8 (5,2–8,4)	237 (122–469)	2.28 (1,48–3,30)	99	15.2	23.1	NA	1,49 (< 0,8)	NC	HC and hypophosphatemia were effectively corrected with phosphate supplementation and did not recur following discontinuation of therapy
9^LV^	2y5m		0.31/0.13	10.3 (8,8–10,8)	4.7 (4,5–6,5)	258 (143–335)	1.86 (1,15–2,44)	95	25.3	16.5	NA	0,07 (< 0,2)	Normal	
10^PV^	6y11m	M/History of neonatal pneumonia- > detected hypercalcemia	− 0,57/− 0,26	10.0 (8,8–10,8)	4.8 (3,6–5,8)	258 (142–335)	1.92 (1,15–2,44)	96	7.2	30.7	26	0,26(< 0,2)	NC	HC resolved with phosphate supplementation at a dose of 5 mg/kg/day
10^LV^	7y5m		− 0.23/− 0.46	10.3 (8,8–10,8)	4.6 (3,6–5,8)	249 (142–335)	1.69 (1,15–2,44)	93	14.5	28.0	NA	0,06(< 0,2)	NC	
11^PV^	5m	F/failure to thrive	< − 2/ < − 2	12.9 (9–10,6)	1.6 (5,2–8,4)	153(122–469)	1.30 (1,48–3,30)	81	1.4	42.8	39.2	1,95(< 0,8)	NC	HC resolved with phosphate supplementation at a dose of 10 mg/kg/day
11^LV^	4y0m		− 1.6/− 0.7	10.2 (8,8–10,8)	4.1 (4,5–6,5)	224(142–335)	NA	NA	20.8	20	NA	0,06(< 0,2)	Normal	

*PV* presentation visit, *LV* last visit, *m* mounts, *y* years, *NA* not available, *F* female, *M* male, *HC* Hypercalciuria, *NC* Nephrocalcinosis, *ALP* high alkaline phosphatase

*, high alp values in follow-ups, sU-Ca/Cre,spot urine calcium creatinine ratio, Numbers in parentheses refer to normal value patients 8 and 9 were previously presented (7)

**Fig. 2:**
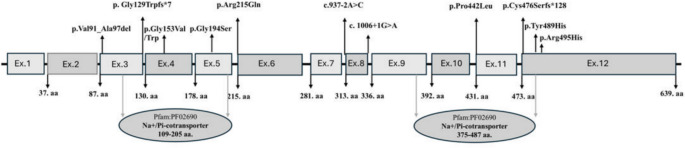
Distribution of diseases causing variants in the context of SLC34A1 gene structures. The Figure shows the linear maps of the gene and the location of the pathogenic mutations. Each rectangle represents a single exon. Ex:Exon, exons are referenced in the ensemble database, Na + /Pi-cotransporter family is referenced in the interpro database

We present longitudinal clinical and laboratory data from 11 patients harboring 12 *SLC34A1* variants, including three novel and nine previously reported mutations, identified across 11 independent families. Seven variants were classified based on ACMG-AMP criteria as pathogenic; three variants were deemed of uncertain significance, while the remaining three were classified as likely pathogenic. All variants, except one (c.272_292del), exhibited extremely low frequency in both gnomAD and the Turkish Variome (PM2). The molecular genetic characteristics of the variants are summarized in Table 2. Figure 2 illustrates the distribution of causative variants within the structural framework of the SLC34A1 gene.

**Table 2:** Summary of genetic variants identified in the patients

Proband	cDNA level	Aminoacid level	Zygosity	Allele frequency*	Segregation analysis mother/father	Phenotype of parent’s mother/father	ACMG evidence	ACMG classification	Novel variant or previously reported variant (PRV)
Family 1	c.1325C > T	p.Pro442Leu	Hom	0.000163/–	Het/Het	Normal/normal	VUS	PM2, PP3	Novel Variant
Family 2	c.1006 + 1G > A	Splicing	Hom	0.000261/0.000298	Het/WT	Normal/normal	Pathogenic	PVS1, PM2, PP5	PRV (21)
Family 3	c.458G > T	p.Gly153val	Het	0.000031/–	NA	The story is not clear	Pathogenic	PM1*, PM2, PM5*, PP3, PP5	PRV (21)
Family 4	c.644G > A	p.Arg215Gln	Hom	0.000141/–	Het/Het	Normal/normal	Pathogenic	PM1*, PM2, PM5, PP3, PP5, BP6	PRV (6)
Family 5	c.383dupc	Gly129Trpfs*7	C.Het	–/–	Het/WT	Nephrolithiasis/nephrolithiasis	Likely Pathogenic	PVS1, PM2	PRV (7)
Family 5	c.937-2A > C	Splicing	C.Het	0.000686/–	WT/Het		Pathogenic	PVS1, PM2, PP5	PRV (7)
Family 6	c.1465 T > C	p.Tyr489His	Het	0.000004/0.000178	Het/Na	Nephrolithiasis/normal	VUS	PM2, PP3	Novel Variant
Family 7	c.1425_1426delCT	p.Cys476Serfs*128	Het	–/–	Het/WT	Nephrolithiasis/normal	Pathogenic	PVS1, PM2, PP5	PRV (21)
Family 8	c.457G > T	p.Gly153Trp	C.Het	–/–	Het/WT	Normal/normal	Pathogenic	PM1*, PM2, PM5*, PP3,	RV (7)
Family 8	c.1484G > A	p.Arg495His	C.Het	0.000123/–	Het/Het	Mother’s uncle: nephrolithiasis	Likely Pathogenic	PS3, PM2, PM3, PP3	PRV (22)
Family 9	c.272_292del	p.Val91_Ala97del	Het	0.0225/0.01	Het/WT	Normal/normalgrandmother: nephrolithiasis	VUS	PM4, PP5, BA1, BS2, BP6	PRV (21)
Family 10	c.580G > A	p.Gly194Ser	Hom	0.000028/0.000534	NA	Nephrolithiasis/normal	Likely Pathogenic	PM2, PM5, PP3	Novel Variant
Family 11	c.1006 + 1G > A	Splicing	Hom	0.000261/0.000298	NA	Nephrolithiasis/normal	Pathogenic	PVS1, PM2, PS4	PRV (21)

*ACMG* American College of Medical Genetics and Genomics, *Het* heterozygous, *Hom* homozygous, *C.HET* compound heterozygous *WT* wild type, *NA* not available -, absent, *VUS* variants of uncertain significance

*gnomad Turkish Variome showed respectively. NM_003052.5 were used for *SLC34A1*

## Discussion

Pathogenic variants in the *SLC34A1* gene give rise to a rare and heterogeneous spectrum of renal tubular disorders, emphasizing the critical importance of specialized clinical expertise for accurate diagnosis and management. While affected individuals may exhibit a range of tubulopathies, hypercalciuria remains a definitive biochemical hallmark. Prevention of nephrocalcinosis and subsequent progression to chronic kidney disease due to persistent hypercalciuria should be considered a primary therapeutic goal [7, 11, 12].

Reduced synthesis or availability of 1,25(OH)₂D₃, in combination with phosphate supplementation, has been associated with a decrease in renal calcifications. Therefore, phosphate supplementation is considered a beneficial therapeutic strategy in children with SLC34A1 mutations [20].

In this study we observed that hypercalciuria resolved in 10 of our patients following phosphate supplementation, irrespective of the presence or absence of baseline hypophosphatemia. The administered doses ranged from 5 to 20 mg/kg/day, providing important clinical insight into long-term therapeutic strategies. In contrast to the observations by Brunkhorst et al. [11], our findings demonstrate the consistent efficacy of oral phosphate therapy in controlling hypercalciuria. This is exemplified in Case 4 in this study. In this patient discontinuation of phosphate treatment led to the recurrence of hypercalciuria despite normal serum phosphate levels, reintroduction of supplementation once again normalized urinary calcium excretion. A patient carrying the same mutation as Case 4 was previously reported in Israel, where the disease progressed to chronic renal failure by age 12 [7]. These observations underscore the utility of oral phosphate therapy as a targeted intervention to mitigate hypercalciuria in *SLC34A1*-related disorders, irrespective of baseline phosphatemia. Consistent with findings from a recently published study we observed that patients harboring biallelic *SLC34A1* variants typically presented at an earlier age, likely due to manifestations of infantile hypercalcemia [11]. However, it is noteworthy that a homozygous individual (Case 10) was diagnosed at the age of five in this study, highlighting the variability in age of onset. As previously reported by Brunkhorst et al., nephrocalcinosis was a universal finding among our cohort, reinforcing the need for early recognition and management. While our biochemical profiles are largely in agreement with those described by Brunkhorst et al. in *SLC34A1* variant carriers, we underscore the potential for progressive clinical heterogeneity over time. This phenotypic variability has also been described as age-dependent phenotypic transitions in affected individuals [21]. During follow-up, patients initially diagnosed with infantile hypercalcemia did not experience recurrent hypercalcemia; however, their serum calcium levels consistently remained at the upper limit of the normal range. We also observed fluctuations in phosphate status, including intermittent episodes of hypophosphatemia, some of which were present at the time of diagnosis. Therefore, the absence of overt tubulopathy at presentation does not exclude the diagnosis of an *SLC34A1*-related disorder. Based on our clinical experience, we propose that the correction of hypercalciuria through oral phosphate supplementation plays a critical role in preserving renal function in affected individuals. We recommend that individuals with a genetically confirmed diagnosis of *SLC34A1* pathogenic variants undergo lifelong surveillance for evolving or latent tubulopathies.

These observations, considered within the context of existing literature, raise the question of whether long-term phosphate supplementation should be continued as a preventive measure against nephrocalcinosis and its potential progression to chronic renal failure, which can occur in an asymptomatic manner.

However, the long-term benefit of phosphate treatment is questioned. Npt2a-/- mice respond differently to dietary phosphate when compared to WT mice and that the degree of renal mineralization positively correlates with serum phosphate [22]. Phosphate supplementation can cause consecutive increase in PTH levels, which may further increase renal phosphate loss and stimulate 1,25-dihydroxyvitamin D3 synthesis, resulting in elevated serum calcium. A resurgence in calcium-phosphorus product levels within the renal tubules can potentially contribute to progressive kidney damage [12]. In our cohort, however, we did not observed such an outcome-even nephrocalcinosis resolved in three patients during follow-up with phosphate supplementation. In a recent study by Brunkhorstet al., the observed elevation in parathyroid hormone (PTH) levels following oral phosphorus supplementation did not reach statistical significance. [11, 12]. It is important to acknowledge that the current evidence regarding the potential risks of phosphorus therapy remains limited.

Consequently, novel therapeutic approaches targeting the underlying molecular mechanisms—such as Burosumab for *PHEX* mutations and IZN-701 for *ENPP1* mutations—are needed. Nonetheless in the current situation, oral phosphate supplementation remains a cornerstone of management [23, 24]. We wish to underscore the increase in height SDS observed in all but one of the patients presented, highlighting the potential effectiveness of oral phosphorus therapy in promoting linear growth. Patients 1,3,4,6 and 7 are still short.

Upon a comprehensive analysis of the genomic locations of the identified variants, we found that nearly all mutations—excluding those in intronic regions—were clustered at or near the Na + /Pi- cotransporter domain. This observation may serve as a key indicator of the functional relevance of this region. Notably, the segments spanning amino acid residues 109–205 and 375–487 within the Na + /Pi- cotransporter represent critical domains of the sodium-dependent phosphate transport protein 2A, encoded by SLC34A1, and warrant further investigation in future functional studies. To our knowledge, this potential hotspot has not been previously highlighted in the literature, underscoring the possibility of novel insights into disease pathogenesis. We identified two intronic mutations, one located at a splice acceptor site and the other at a splice donor site, both of which are predicted to have a high likelihood of pathogenicity and were accordingly classified as pathogenic under ACMG guidelines. An intriguing observation was that 11 of the 13 intronic mutations reported in the ClinVar database were similarly positioned, residing one or two nucleotides from exon–intron boundaries—regions critical for proper splicing. We also detected two distinct variants affecting codon 153, one of which was heterozygous in our patient and previously described in the biallelic state as disease-causing [11, 25]. The other variant at this codon was observed in a compound heterozygous context, paired with a missense mutation at codon 495, and was similarly associated with disease[26]. Notably, three different amino acid substitutions at the Gly153 residue—including the novel Trp substitution reported in this study—have been implicated in pathogenicity, suggesting that Gly153 is a functionally critical and mutationally sensitive residue [25]. A similar pattern was observed for the novel p.Gly194Ser variant, which lies within the Na + Pi- cotransporter domain. The ClinVar database documents a different substitution at the same residue, p.Gly194Arg, as pathogenic, reinforcing the likely functional importance of this site. Additionally, we report a single-nucleotide substitution (guanine to adenine) at the 644th nucleotide, which is the terminal base of exon 5, resulting in a missense change at amino acid position 215 (p.Arg215Gln). Given that mutations at the last nucleotide of exons are known to disrupt normal splicing [27], this variant satisfies the PM1 criterion of the ACMG classification system and was thus deemed pathogenic. Lastly, some of our monoallelic patients may harbor additional potentially pathogenic variants in other genes that have a role in phospahate homeostatsis in an oligogenic model [28]. Two of The three novel variants we reported were classified as VUS, but due to the clinical presentation of infantile hypercalcemia in the cases, we considered these variants to be the cause of the disease. However, we would like to point out that functional analyses of these novel variants are not available. Collectively, these findings emphasize the importance of mapping variant locations within the protein’s functional domains and splicing-relevant regions, as such positional data can significantly inform pathogenicity assessments and clinical interpretation.

## Conclusion

Through the identification of 12 distinct *SLC34A1* mutations, including three novel variants—in 11 unrelated cases, this study broadens the current understanding of the genetic spectrum associated with this disorder. The findings offer valuable insights into the clinical heterogeneity of the disease, underscoring the need for heightened awareness of atypical or subclinical manifestations that may otherwise be underdiagnosed or inadequately monitored. The distribution of the detected mutations highlights the essential functional importance of the Na + Pi- cotransporter domain. Moreover, we emphasize the therapeutic value of oral phosphate supplementation in mitigating long-term complications associated with hypercalciuria, a frequent and clinically significant feature in patients with *SLC34A1*-related disorders.

## Data Availability

All data generated or analyzed during this study are included in this article. Further enquiries can be directed to the corresponding author.
